# Body composition and personality traits in so-Yang type males

**DOI:** 10.1186/s12906-017-1919-5

**Published:** 2017-08-22

**Authors:** Jiwon Yoon, Jiho Nam, Chae Hun Leem, Jong Yeol Kim

**Affiliations:** 1 KM Fundamental Research Division, Korea Institute of Oriental Medicine, 1672 Yuseong-daero, Yuseong-gu, Daejeon, 34054 Republic of Korea; 2 Department of Physiology, University of Ulsan College of Medicine, 88 43-Gil Olympic-Ro, Songpa-gu, Seoul, 05505 Republic of Korea

**Keywords:** Body composition, Skeletal muscle mass, Sasang constitutional medicine, Personality

## Abstract

**Background:**

The purpose of the present study was to examine the body composition of So-Yang type males according to Sasang constitutional medicine, which is popular in Korea. Different Sasang constitutional types are associated with different muscle distributions, body shapes, and disease susceptibilities. We used the Sasang Personality Questionnaire (SPQ) as a measure of the temperament of each Sasang type.

**Methods:**

In total, 953 subjects aged over 20 years were recruited in Korea. We collected anthropometric parameters and bioimpedence information from the subjects and administered the SPQ. A logistic regression was conducted to calculate propensity scores.

**Results:**

The percentage of skeletal muscle mass in So-Yang (SY) and non-So-Yang (non-SY) males was 45.8 ± 2.7 and 44.2 ± 3.3, respectively, before matching and 45.8 ± 2.6 and 44.9 ± 3.0, respectively, after propensity score matching. The extracellular water (ECW)/intracellular water (ICW) and extracellular water (ECW)/total body water (TBW) ratios and SPQ scores were significantly different between the SY and non-SY types.

**Conclusions:**

This study suggested that the SY type may be significantly and independently associated with body composition and could be associated with personality.

## Background

In recent years, several studies have shown a relationship between high body mass index (BMI) and metabolic syndrome [1]. However, not all obese individuals shows evidence of metabolic syndrome [2, 3]. Thus, there have been several attempts to identify a correlation between body shape and metabolic risk factors. Typically, people who have metabolic syndrome have been characterized as having apple-shaped (abdominal fat) bodies rather than pear-shaped (peripheral fat) bodies [4]. In contrast to peripheral fat mass, visceral fat mass is strongly associated with obesity-related complications such as cardiovascular disease and Type 2 diabetes [5]. Additionally, waist circumference and the waist-to-hip ratio are used to predict metabolic syndrome [6]. Recently, the existence of a metabolically obese, normal weight phenotype associated with normal weight and obese characteristics was proposed [7, 8]. Even with a low BMI, there is a high risk for cardiovascular disease in people with a high body fat percentage [9, 10]. The BMI, waist circumference, and body fat percentage are focused solely on body fat mass and do not take into account the body muscle mass, which accounts for 40–50% of the body composition. Among body muscle mass, skeletal muscle plays a crucial role in glucose metabolism and insulin resistance [11]. Recent studies have shown correlations between low muscle mass and risk factors for cardiovascular disease such as diabetes, hypertension, and dyslipidemia [12]. A decline in muscle mass has been reported as a risk factor for the development of metabolic syndrome [13]. Moreover, relative muscle loss is associated with insulin resistance and the risk of diabetes [14].

In Korea, Sasang constitutional medicine (SCM) was proposed by Lee Je-ma in 1894. According to his theory, humans can be classified into four typologies, Tae-Yang (TY), Tae-Eum (TE), So-Yang (SY), and So-Eum (SE), which are based on characteristics of temperament, body shape and physiology. SCM has been developed and widely used in clinical traditional Korean medicine and the proportion of constitutional medical service in the Korean medical service market was 23.5% [15–17]. During the last ten-year period, scientific research on SCM has been published in medical journals and actively and vibrantly discussed. One notable observation regarding SCM research is that inheritable aspects of SCM have been investigated through genetic polymorphisms [18–20]. In particular, SCM focuses on the relationship between anthropometric factors, temperament, and disease susceptibility. With regard to constitution, each typology exhibits a different, unique susceptibility to particular disease, indicating that medication should be prescribed based on each individual’s Sasang type.

Research on certain types of inherited constitutional body types was categorized by Sheldon. Sheldon categorized humans into three different types: components: endomorph (fatness), mesomorph (musculoskeletal robustness), and ectomorph (linearity). Regarding the association with SCM, endomorph, mesomorph and ectomorph are considered to be closely related to the TE, SY, and SE types, respectively [21]. Recently, many studies have explored the relationship between somatotypes and sports ability from diverse angles. According to those results, the mesomorphic component is dominant in certain types of elite players [22–27]. Additionally, there have been attempts to identify relationships between somatotypes and disease susceptibility. For example, the endomorphic component is related to high blood pressure [28, 29], abdominal obesity [30], coronary artery disease [30], and type 2 diabetes mellitus [31].

Like somatotypes, there have been various studies investigating the association of SCM with BMI [32], abdominal obesity [33], and disease susceptibility [34, 35]. Based on the previous reports, the prevalence and relative risk of obesity [33], hypertension [34], diabetes mellitus [35, 36], and metabolic syndrome [37] are higher in the TE type compared with other types. The SE type tends to have weak digestive function and a higher frequency of digestive disease [38–40]. However, few studies have been performed on the SY type and disease susceptibility. Relative muscle mass is inversely associated with insulin resistance and plays a key role in maintaining physical ability [14, 41]. Based on these results, it can be assumed that there is a relationship between muscular percentage and the SY type’s relatively healthy condition. Thus, in this study, we focused on body fat percentage and skeletal muscle percentage assess the characteristics of the SY type individuals.

## Methods

### Subjects

This cross-sectional study was conducted from 2009 to 2015. All of the questionnaire data and clinical data, including Sasang constitutional types (SCTs), were compiled from the Korea Constitutional Multicenter Bank (KCMB) of the Korea Institute of Oriental Medicine (KIOM). Using this resource, we collected questionnaire data on 953 adults (20 years old or older) who were admitted to the Asan Medical Center. The present study population comprised a population-based sample selected from healthy subjects recruited through advertisements at Asan medical center. To eliminate potential effects of metabolic syndrome on the analysis, we excluded participants with metabolic syndrome. Metabolic syndrome was defined as when three or more of the following conditions were present: high triglyceride levels (≥150 mg/dl), low high-density lipoprotein cholesterol (<40 mg/dl in men, <50 mg/dl in women), high blood pressure (≥130/85 mmHg), high fasting blood glucose (≥100 mg/ml), and abdominal obesity (waist circumference (≥90 cm in men, ≥80 cm in women). Individuals who could not be categorized to identify their SCT, were excluded. After applying these exclusions, a total of 567 individuals were analyzed. This study was approved by the Institutional Review Board of the Asan Medical Center. All participants agreed to join this study, and written informed consent for participation was obtained from every subject.

### Data collection

Blood pressure were measured by standard methods. The blood pressure was measured at rest in the left upper arm. Blood Samples were obtained from the left brachial vein after more than 12 h of fasting. Triglyceride (TG), high-density lipoprotein (HDL) cholesterol, and fasting blood glucose levels were measured in a central laboratory.

### Sasang constitutional diagnosis

An integrated diagnostic model developed in a previous study, namely the Sasang Constitutional Analysis Tool (SCAT), was used to classify the subjects into different SCTs based on the probability values for each type [42]. Four individual quantitative data sets, such as facial images, body shapes, voice analysis, and questionnaire responses on personality and physiological symptoms were integrated into a single value SCAT. Once individual levels of all required items were entered into the SCAT system, it presented the percentage of each person’s potential to be categorized as a SCT using a multinomial logistic regression analysis. Then, a person’s SCT with the highest percentage is determined as the person’s final type. The diagnostic accuracy of SCAT was higher than that of QSCC II, which has been commonly used for the classification of SCTs and is widely used in previous researches [36, 43]. Briefly, the facial images of subjects that were obtained with a digital camera were appropriately processed to extract variables for facial points and contours. Variables for facial points and contours include the following: width, height, areas, angle and depth, and ratio of face shape, forehead, eye, upper eyelid, and nose. For the body shape analysis, the following 11 variables were collected: forehead circumference, neck circumference, axillary circumference, chest circumference, rib circumference, waist circumference, pelvic circumference, hip circumference, height, weight and body mass index (BMI). Body measurement data were collected from each subject while wearing light clothing. A subject maintains stable breathing and measurement is made between inspiration and expiration. Height and body weight were measured to the nearest 0.1 cm and 0.1 kg, respectively. BMI was computed as weight divided by height squared (kg/m^2^). 8-circumference indices were measured by well-trained observers using standard operating procedures developed for the Korea Constitution Multicenter Study. The circumferences of eight regional sites of the body are measured at the levels of the glabella and opisthion (forehead circumference), the thyroid cartilage and cricoid cartilage (neck circumference), the left and right axilla (axillary circumference), the left and right nipple points (chest circumference), the left and right 7th and 8th prominence of costochondral junction (rib circumference), the umbilical cord (waist circumference), the left and right anterior superior iliac spines (pelvic circumference), and the upper edge of the pubis (hip circumference). Voice analysis was performed using the Hidden Markov Model Toolkit (HTK) and Praat voice-analysis programs. A voice signal with a minimum duration of 40 ms was selected for feature extraction. More than 200 features from the vowels and the sentences were extracted as an initial set, and 88 features were finally selected for a final diagnostic model after applying a genetic algorithm-based feature selection technique The questionnaire for SCTs consisted of 67 multiple-choice questions that included personality characteristics, general temperament, eating habits (e.g., whether having regular meals, frequency of eating meals a day and eating speed), and physiological symptoms (e.g., perspiration, excrement, discomfort in the body, location of discomfort during illness, and existence of fatigue). Sasang constitution questionnaire was developed by KIOM standardized and validated in 2009 and demonstrated good reliability [44, 45]. Cronbach’s alpha was 0.801 in the personality index and Cronbach’s alpha was 0.598 in the case of the physiological symptoms index [46]. Thus, the internal consistency of the questionnaire was confirmed.

### Bioimpedence assessments

Skeletal muscle mass, body fat mass, and weight were measured by multi-frequency bioelectric impedance analysis (Inbody 770, eight-point tactile electrode methods, Biospace Co. Ltd., Seoul, Korea). This system uses an electrical current at different frequencies (5, 50, 250, 500, and 1000 kHz) to directly measure the amount of extracellular and intracellular water in the body. Four electrodes were placed on the palm and thumb of both hands, and four electrodes were placed on the anterior and posterior aspects of the soles of both feet. Based on these impedance values, skeletal muscle mass (SMM) (kg), body fat mass (BFM) (kg), intracellular water (ICW) (kg), extracellular water (ECW) (kg) and total body water (TBW) (kg) were calculated. Body composition parameters based on these variables were defined as follows, to estimate not only the effect of absolute mass but also the effect of the relative ratio on body composition.

SMM (kg) was converted to SMM percentage (skeletal muscle mass/body weight × 100)

BFM (kg) was converted to BFM percentage (body fat mass/body weight × 100)

### Sasang personality questionnaire

The Sasang Personality Questionnaire (SPQ) is a 14-item self-report questionnaire developed for the measurement of psychological traits of the Sasang constitutions. The SPQ has the following three personality subscales: SPQ-Behavior (SPQ-B), SPQ-Emotion (SPQ-E), and SPQ-Cognition (SPQ-C). The SPQ-B measures the behavioral component (passive vs. active); the SPQ-E measures the emotional level (static vs. dynamic); and the SPQ-C measures cognition and decision making (meticulous vs. easy going). The SPQ score is the total score of the components of these three subscales. Its structural validity and clinical reliability have been reported [47, 48], and the internal consistency values of the SPQ, SPQ-B, SPQ-C, and SPQ-E were 0.81, 0.74, 0.62, and 0.62 [47], respectively. The total SPQ score and the scores of each subscale were found to increase in order from the SE to TE to SY Sasang types [47, 48].

### Statistical analysis

**Fig. 1:**
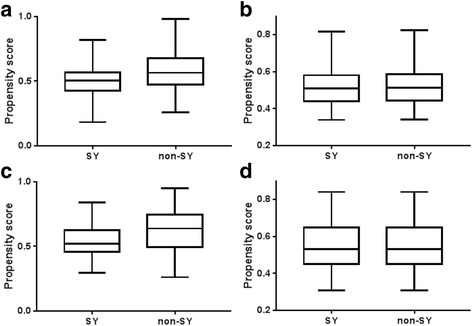
Comparision of the propensity scores between the SY and non-SY types before and after propensity matching: **a** Male propensity score before matching, **b** Male propensity score after matching, **c** Female propensity score before matching, and **d** Female propensity score after matching. SY: So-Yang; non-SY: Tae-Eum and So-Eum

All statistical analyses were performed using SPSS version 23.0 (SPSS, Chicago, IL, USA). All *p*-values <0.05 were considered statistically significant. TY type subjects were unavoidably excluded due to the extremely small sample size of the TY type. Since the population of TY is extremely low, SY can represent Yang group and TE and SE can be categorized into same Yin group. Subjects in the SY and non-SY groups were matched 1:1 using propensity scores. The physical characteristics were matched using a propensity score consisting of age, height, weight, and BMI. We calculated the propensity score for each SY and non-SY type using multivariate logistic regression in males and females separately. A matching process was conducted with a minimum distance scoring method, and each propensity score of a SY type was matched with the closest propensity score of a non-SY type. Specifically, matching was limited to a caliper width of 0.05. Figure 1 shows the change in propensity score distribution between the matched SY and non-SY groups. Data are expressed as the means ± standard deviations. Between-group comparisons were performed using the independent-samples *t*-test.

## Results

### General characteristics of the study population

Table 1 shows the general characteristics of the participants enrolled in this study. The number of subjects in the original SY and non-SY groups was 126 and 150 in males and 119 and 172 in females, respectively. The general characteristics such as weight and BMI were significantly different between the SY and non-SY types before matching (*p* < 0.001). After propensity score matching, the number of SY and non-SY subjects in each group was 94 males and 97 females, with no statistically significant differences in the general characteristics between the groups.

**Table 1:** General characteristics according to the SC before and after propensity score matching

Variables	Before matching			After matching		
	SY type	non-SY type	p	SY type	non-SY type	p
Male						
n	126	150		94	94	
Height (cm)	172.4 ± 5.7	173.4 ± 5.6	0.132	172.4 ± 5.8	172.0 ± 5.5	0.641
Weight (kg)	69.3 ± 6.6	73.5 ± 11.4	<0.001	69.4 ± 6.7	69.2 ± 8.3	0.805
BMI (kg/m2)	23.3 ± 1.8	24.4 ± 3.2	<0.001	23.3 ± 1.8	23.4 ± 2.6	0.935
Age (yrs)	34.6 ± 11.1	36.5 ± 11.5	0.344	35.9 ± 11.6	37.6 ± 11.4	0.310
SBP (mmHg)	114.4 ± 13.6	114.6 ± 12.7	0.891	114.8 ± 13.9	114.2 ± 12.6	0.736
DBP (mmHg)	68.8 ± 9.4	69.6 ± 9.2	0.462	69.4 ± 9.6	69.1 ± 8.7	0.842
Fasting Glucose (mg/dL)	95.2 ± 10.1	97.4 ± 15.7	0.197	95.9 ± 10.8	95.7 ± 12.9	0.909
TG (mg/dL)	107.1 ± 98.6	113.1 ± 62.4	0.544	114.2 ± 112.5	103.5 ± 42.8	0.406
HDL-C (mg/dL)	54.4 ± 13.1	51.7 ± 12.0	0.078	54.6 ± 13.0	53.1 ± 11.3	0.402
WC (cm)	74.0 ± 9.6	76.0 ± 9.6	0.062	75.0 ± 8.4	74.9 ± 8.3	0.951
Female						
n	119	172		97	97	
Height (cm)	158.8 ± 5.2	160.5 ± 5.4	0.010	159.0 ± 5.4	159.3 ± 5.1	0.717
Weight (kg)	53.9 ± 4.1	57.5 ± 8.8	<0.001	54.3 ± 4.1	54.3 ± 7.1	0.960
BMI (kg/m2)	21.4 ± 1.6	22.3 ± 3.3	0.001	21.5 ± 1.7	21.4 ± 3.0	0.908
Age (yrs)	36.5 ± 11.1	40.3 ± 11.8	0.005	37.2 ± 11.4	37.3 ± 10.9	0.943
SBP (mmHg)	102.7 ± 11.8	105.9 ± 12.2	0.024	103.5 ± 12.0	103.3 ± 12.0	0.915
DBP (mmHg)	62.5 ± 7.6	65.0 ± 8.2	0.008	62.8 ± 7.6	64.0 ± 7.6	0.250
Fasting Glucose (mg/dL)	89.4 ± 13.2	91.9 ± 11.5	0.091	89.6 ± 14.5	91.1 ± 11.6	0.450
TG (mg/dL)	76.2 ± 32.3	83.1 ± 35.5	0.096	77.7 ± 33.5	78.2 ± 30.5	0.923
HDL-C (mg/dL)	64.8 ± 14.7	63.2 ± 12.7	0.346	64.7 ± 14.4	62.7 ± 11.9	0.298
WC (cm)	76.2 ± 5.8	80.4 ± 8.7	<0.001	76.4 ± 5.9	77.9 ± 8.2	0.154

Data shown are the means ± SDs or numbers. After matching, the data shown are the means ± SDs. *SC* sasang constitution, *SY* so-yang, non-SY: Tae-Eum and So-Eum; *BMI* body mass index, *SBP* systolic blood pressure, *DBP* diastolic blood pressure, *TG* triglyceride, *HDL-C* high-density lipoprotein cholesterol, *WC* waist circumference

### Body composition index

Gender-specific values for the body composition index are shown in Table 2. Because non-SY types had significantly increased weight and BMI as shown in Table 1, general body composition values (BFM, SMM, ICW, ECW, and TBW) were higher in the non-SY types compared with the SY type. However, when converted to body weight, both males and females with the SY type had a significantly (*p* < 0.001) greater SMM percentage. These differences remained significant (*p* = 0.021) after propensity matching for age, height, weight and BMI in males. The BFM percentage was lower in the SY group in both males and females, but after propensity matching, this difference became insignificant. Compared to the non-SY types, both males and females with the SY type had significantly lower ECW/ICW and ECW/TBW ratios before and after propensity matching.

**Table 2:** Body composition index according to the SC before and after propensity matching

Variables	Before matching			After matching		
	SY type	non-SY type	p	SY type	non-SY type	p
Male						
BFM (kg)	13.2 ± 3.6	16.2 ± 6.4	<0.001	13.1 ± 3.6	14.1 ± 4.7	0.115
SMM (kg)	31.7 ± 3.2	32.3 ± 4.2	0.165	31.8 ± 3.1	31.0 ± 3.5	0.093
BFM percentage (%)	18.9 ± 4.5	21.5 ± 5.9	<0.001	18.7 ± 4.3	20.0 ± 5.2	0.057
SMM percentage (%)	45.8 ± 2.7	44.2 ± 3.3	<0.001	45.8 ± 2.6	44.9 ± 3.0	0.021
ICW	25.8 ± 2.4	26.3 ± 3.2	0.161	25.9 ± 2.3	25.3 ± 2.7	0.098
ECW	15.4 ± 1.4	15.8 ± 1.8	0.055	15.5 ± 1.4	15.2 ± 1.5	0.200
TBW	41.2 ± 3.8	42.1 ± 5.0	0.109	41.4 ± 3.7	40.5 ± 4.2	0.126
ECW/ICW	0.597 ± 0.015	0.601 ± 0.014	0.028	0.598 ± 0.015	0.602 ± 0.014	0.048
ECW/TBW	0.374 ± 0.006	0.375 ± 0.006	0.028	0.374 ± 0.006	0.376 ± 0.005	0.047
Female						
BFM (kg)	15.0 ± 3.1	17.5 ± 5.9	<0.001	15.1 ± 3.1	15.8 ± 5.0	0.264
SMM (kg)	21.1 ± 2.0	21.6 ± 2.6	0.062	21.2 ± 2.0	20.8 ± 2.3	0.159
BFM percentage (%)	27.1 ± 5.6	29.8 ± 6.3	<0.001	27.0 ± 5.9	28.6 ± 5.9	0.077
SMM percentage (%)	38.8 ± 4.6	37.5 ± 5.3	0.021	38.7 ± 4.9	38.1 ± 5.1	0.363
ICW	17.7 ± 1.5	18.1 ± 2.0	0.068	17.8 ± 1.6	17.5 ± 1.7	0.152
ECW	10.9 ± 1.0	11.2 ± 1.2	0.006	10.9 ± 1.0	10.9 ± 1.1	0.546
TBW	28.6 ± 2.5	29.3 ± 3.2	0.028	28.8 ± 2.5	28.3 ± 2.8	0.260
ECW/ICW	0.615 ± 0.013	0.622 ± 0.013	<0.001	0.615 ± 0.013	0.622 ± 0.013	<0.001
ECW/TBW	0.381 ± 0.005	0.383 ± 0.005	<0.001	0.381 ± 0.005	0.383 ± 0.005	<0.001

Data shown are the means ± SDs. After matching, the data shown are the means ± SDs. *SC* sasang constitution, *SY* so-yang, *non-SY* Tae-Eum and So-Eum, *BFM* body fat mass, *SMM*: skeletal muscle mass *ICW* intracellular water, *ECW* extracellular water, *TBW*, total body water

### SPQ scores

The SPQ-B, SPQ-E, and SPQ-C scores were found to be significantly different between types. A t-test was conducted to examine differences between the SY and non-SY types. Before matching, the SPQ-B, SPQ-E, and SPQ-C scores of the SY type (12.01 ± 2.31, 7.82 ± 1.99, and 10.25 ± 1.81, respectively) were significantly higher than those of the non-SY types (10.10 ± 2.41, 7.12 ± 2.00, and 9.69 ± 2.05, respectively), in males (Fig.﻿ 2﻿a﻿). After propensity score matching, the SPQ-B, SPQ-E, and SPQ-C scores of the SY type (11.96 ± 2.31, 7.84 ± 1.90, and 10.21 ± 1.82) were still significantly higher than those of the non-SY types (9.89 ± 2.34, 6.94 ± 1.99, and 9.42 ± 1.99, respectively), in males (Fig.﻿ 2﻿b﻿﻿). Before matching, the SPQ-B, SPQ-E, and SPQ-C scores of the SY type (11.96 ± 2.05, 8.75 ± 1.86, and 10.61 ± 2.04, respectively) were significantly higher than those of the non-SY types (10.29 ± 2.60, 7.67 ± 2.13, and 9.20 ± 2.28, respectively), in females (Fig.﻿ 2c﻿). After propensity score matching, the SPQ-B, SPQ-E, and SPQ-C scores of the SY type (11.98 ± 2.02, 8.70 ± 1.90, and 10.65 ± 2.16, respectively) were still significantly higher than those of the non-SY types (10.14 ± 2.67, 7.65 ± 2.02, and 9.18 ± 2.40, respectively), in females (Fig.﻿ 2﻿d).

**Fig. 2:**
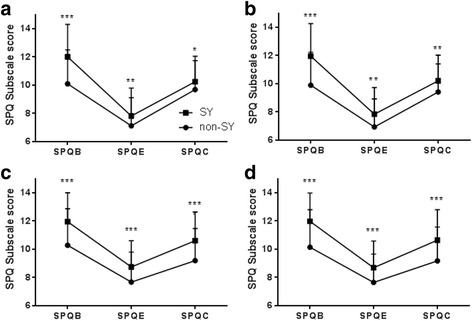
The SPQ subscale scores of the SY and non-SY types. **a** The SPQ subscale scores of the male subjects before propensity score matching, **b** the SPQ subscale scores of male subjects after propensity score matching, **c** the SPQ subscale scores of female subjects before propensity score matching, and **d** the SPQ subscale scores of female subjects after propensity score matching. SPQ: Sasang Personality Questionnaire; SPQB: SPQ behavior; SPQE: SPQ emotion; SPQC: SPQ cognition; SY: So-Yang, non-SY: Tae-Eum and So-Eum

## Discussion

Previous research demonstrated differences in the physical capacities of different Sasang constitutions. Ko et al. reported that the ability to convert energy into muscle strength and speed is greater in the SY type than in other types [49]. Pham et al. indicated that maximal oxygen consumption (VO_2max_, mL∙kg^−1^∙min^−1^), a measure of cardiorespiratory fitness is highest among SY type males [50]. He also suggested that increased BMI in the SY type is related to elevated energy expenditure but not to reduced physical endurance [51]. We assumed that the physical capacity and cardiorespiratory fitness of the SY type is associated with body composition. Additionally, the SY body composition may be associated with biopsychological level. In summary, SY males had a relatively high percentage of skeletal muscle mass and low ECW/ICW and ECW/TBW ratios compared with the other types, with this tendency maintained after propensity score matching.

The general characteristics of subjects differed markedly depending on height, weight, BMI and age. Because body composition is affected by age and weight, we concluded that these differences could generate bias in the groups. Therefore, the subjects were matched in the SY and non-SY groups using the propensity score matching method (94 male subjects, 97 female subjects). After matching, there was no difference in height, weight, BMI, and age. This meant that selection bias were minimized between SY and non-SY groups and all groups became almost homogeneous.

Many studies have been performed on body composition of Sasang constitutions using the bioelectric impedance analysis method. However, the majority of them focused on body fat percentage, which has consistently been reported to be greater in the TE type [36, 49–52]. The TE type tends to have a higher BMI; therefore, the lean body mass of the TE type has been reported to be greater than that of other types [36, 49–51]. These findings are consistent with the expectation that a heavier person requires greater muscle mass for movement and has more muscle than a lean person [53]. Thus, we converted absolute skeletal muscle mass (kg) to percentage skeletal muscle mass (skeletal muscle mass/body weight × 100). The SMM percentage was high in the SY male group only. Skeletal muscle plays an important role in physical strength, stamina, and balance [54]. Moreover, a low percentage of skeletal muscle mass is related to insulin resistance, diabetes, and metabolic syndrome [13]. In accordance with these results, the present study demonstrates that SY males have excellent body composition and skeletal muscle distribution. This reinforces the hypothesis that physical capacity and aerobic fitness of the SY type is associated with body composition.

The ECW/ICW and ECW/TBW ratios of the SY type were lower than those of the non-SY types. Compared with obese groups, relatively low ECW/ICW and ECW/TBW hydration ratios were reported in lean groups [55]. Low ECW/ICW and ECW/TBW ratios for the SY type reflects a significantly higher percentage of fat free mass of the SY type. These results are consistent with the finding that the ECW/ICW ratio of adipocytes is greater than that of fat free mass cells [56]. In this study, low ECW/ICW and ECW/TBW ratios of the SY type were maintained even after controlling BMI and age. Therefore, it is a strong indication that SY type shows a unique characteristic of body fluid distribution.

Studies have generally shown personality, especially extraversion, to be positively associated with physical activity [57]. Moreover, physical activity increases the development and maintenance of lean muscle mass [58]. Personality is correlated with muscle strength and is mediated by physical activity level [59]. Because the theological basis of SCM depends on insight into the variation in human psychological differences, it can be suggested that personality traits play an important role in an individual’s muscle mass percentage. Therefore, we analyzed the SPQ according to type to assess the presence of such tendencies. The SPQ is a 14-item self-report survey tool used to measure physiological characteristics from the perspective of the SCM. The SPQ consists of three subscales that measure behavioral (SPQ-B), emotional (SPQ-E), and cognitive (SPQ-C) characteristics of personality. The SY type showed a personality profile of active (behavior), dynamic (emotionality), and easy-going (cognition) personality components, with a high overall SPQ score. In contrast, the non-SY types scored relatively lower on the SPQ, with passive (behavior), static (emotionality), and meticulous (cognition) personality components. This finding was consistent with previous studies [47, 60, 61] that SY type repeatedly showed higher score than non-SY type.

Chae et al. noted that the SY type tends to be more extraverted than other constitutions and linked the variation in personality traits to the dopamine system [52]. Based on the Braverman nature assessment, the SY type was reported to have a close relationship to the dopamine system [62]. Dopamine functioning is known to play an important role in voluntary exercise, spontaneous physical activity [63], and extraversion through reward-seeking behavior [64, 65]. This finding provides evidence that the association between the extraverted, active, and distinctive body composition profiles of the SY type is due to dopamine function. Thus, these results open up the possibility that biopsychological feature of different SCTs type may influence physical characteristics of SCTs.

This study has several limitations. First, the classification of SCTs principally depends on the variation of psychological diversities. However, we only used SPQ scale as a measurement of psychological differences and it should be replicated again using other objective scale in order to be a comprehensive, multifaceted approach.. Second, we did not control for exercise habits or physical activity level. Third, we did not measure muscle strength, which is known to be a better predictive factor for physical performance than muscle mass [66]. Further investigations aimed at assessing the relationship between the high percentage of muscle mass of the SY type and other physical and physiological characteristics are needed.

## Conclusions

We found that the percentage of skeletal muscle mass was high in SY males. Moreover, these traits can explain the low ECW/ICW and ECW/TBW ratios of SY males. The high SPQ score of SY males can explain the difference between the SY type and other constitutions.

## Abbreviations

BFM: Body fat mass
BMI: Body mass index
ECW: Extracellular water
ICW: Intracellular water
non- SY: Non-So-Yang
SCM: Sasang constitutional medicine
SMM: Skeletal muscle mass
SPQ: Sasang Personality Questionnaire
SY: So-Yang
TBW: Total body water
